# MicroRNA-125a-5p regulates the effect of Tregs on Th1 and Th17 through targeting ETS-1/STAT3 in psoriasis

**DOI:** 10.1186/s12967-023-04427-6

**Published:** 2023-09-29

**Authors:** Kexiang Yan, Fuxin Zhang, Jie Ren, Qiong Huang, Nikhil Yawalkar, Ling Han

**Affiliations:** 1 Department of Dermatology, Huashan Hospital, Shanghai Institute of Dermatology, Fudan University, Shanghai, 200040 China; 2 Department of Dermatology, Inselspital, Bern University Hospital, University of Bern, Bern, Switzerland

**Keywords:** Psoriasis, miR125a-5p, Tregs, Th17, ETS-1, STAT3

## Abstract

**Background:**

Psoriasis is an inflammatory disease mediated by helper T (Th)17 and Th1 cells. MicroRNA-125a (miR-125a) is reduced in the lesional skin of psoriatic patients. However, the mechanism by which miR-125a participates in psoriasis remains unclear.

**Methods:**

The levels of miR-125a-5p and its downstream targets (ETS-1, IFN-γ, and STAT3) were detected in CD4^+^ T cells of healthy controls and psoriatic patients by quantitative real-time PCR (qRT-PCR). In vitro, transfection of miR-125a-5p mimics was used to analyze the effect of miR-125a-5p on the differentiation of Th17 cells by flow cytometry. Imiquimod (IMQ)-induced mouse model was used to evaluate the role of upregulating miR-125a-5p by intradermal injection of agomir-125a-5p in vivo.

**Results:**

miR-125a-5p was downregulated in peripheral blood CD4^+^ T cells of psoriatic patients, which was positively associated with the proportion of regulatory T cells (Tregs) and negatively correlated with the Psoriasis Area and Severity Index (PASI) score. Moreover, the miR-125a-5p mimics promoted the differentiation of Tregs and downregulated the messenger RNA (mRNA) levels of ETS-1, IFN-γ, and STAT3 in murine CD4^+^ T cells. Furthermore, agomir-125a-5p alleviated psoriasis-like inflammation in an IMQ-induced mouse model by downregulating the proportion of Th17 cells.

**Conclusions:**

miR-125a-5p may have therapeutic potential in psoriasis by restoring the suppressive function of Tregs on Th17 cells through targeting STAT3, and on Th1 cells indirectly through targeting ETS-1 and IFN-γ.

**Supplementary Information:**

The online version contains supplementary material available at 10.1186/s12967-023-04427-6.

## Background

Psoriasis is a chronic, recurrent, inflammatory disease that is mediated by a combination of genetic and environmental factors, affecting approximately 0.09–5.1% of the world’s population [1, 2]. The disease has a strong T cell-mediated immunological component and is primarily driven by dysregulation of T cell subpopulations, including helper T (Th) 17, Th1 cells, and regulatory T cells (Tregs) [3–5]. Our preliminary research has shown that patients with psoriasis have functional defects in Tregs, resulting in insufficient suppression of inflammatory cytokines secreted by Th17 cells and leading to an altered Th17/Treg balance [3, 6–9].

MicroRNAs (miRs) are a class of highly conserved small non-coding RNAs, approximately 22 nt in length, that function as post-transcriptional regulators of gene expression by binding to 3′ untranslated regions (3′ UTRs) of a target messenger RNA (mRNA) and have been associated with psoriasis pathogenesis [10]. miR-125a belongs to the microRNA-125 (miR-125) family, which includes miR-125a and miR-125b. Among these, miR-125a is located on chromosome 19 and contains two different mature miRNAs derived from the 5′ or 3′ arms of the pre-miRNA (5p and 3p) [11]. It has been reported that miR-125a-5p participates in the development and function of immune cells, making it involved in the pathogenesis of autoimmune diseases [12]. Compared with controls, miR-125a-5p deficient mice had more severe colitis [13]. Additionally, miR-125a-5p deficient mice were more prone to developing experimental encephalomyelitis, an autoimmune disease where Tregs cannot suppress Th1 and Th17 cells. Furthermore, the expression of miR-125a-5p was reduced in patients with systemic lupus erythematosus (SLE) [14].

E26 transformation specific-1 (ETS-1), and signal transducer and activator of transcription 3 (STAT3) are transcription factors that have been confirmed as downstream targets of miR-125a-5p in autoimmune diseases [15, 16]. ETS-1 has been found to play a crucial role in promoting the production of interferon-gamma (IFN-γ), and facilitating Th1 cells differentiation [17]. On the other hand, STAT3 directly activates key genes including the promoters of *IL-17 A* and *IL-17 F*, thereby regulating the differentiation of Th17 cells [18]. ETS-1, STAT3, and IFN-γ are important regulators involved in the differentiation of Th1 and Th17 cells, and their relationship with psoriasis and miR-125a-5p remains to be elucidated.

Growing evidence has shown that miR-125a is involved in the pathogenesis of psoriasis. miR-125a expression in psoriatic lesions has been reported to correlate with disease severity, and overexpression of miR-125a could suppress keratinocyte proliferation [15]. Pei et al. reported that plasma miR-125a-5p levels were lower in psoriatic patients than in healthy controls and increased with treatment [19]. In this study, we investigated the expression of miR-125a-5p in CD4^+^ T cells of psoriatic patients and its role in regulating T cell subsets.

## Methods

### Collection of blood samples and isolation of human PBMCs

Blood samples were collected from 30 psoriasis patients and 30 sex-matched healthy donors at the Dermatology Department of Huashan Hospital, Fudan University, Shanghai, China. Before the blood was taken, informed consent was obtained from each patient and oral informed consent records were kept on file. Patient characteristics are available in Additional file 1: Table S1. To be eligible for the study, participants had to meet the following recruitment criteria: they could not have received any systematic treatment for at least 4 weeks before entering the study; conditions such as significant infection and immune suppression were excluded; and participants with significant renal, hepatic, or other diseases were also excluded. The institutional review board protocols of Huashan Hospital, Fudan University (#2021 M-018) approved the study as human subject research.

### IMQ-induced psoriasis-like mouse model

Six-week-old male mice (BALB/C) were purchased from Biotechnology Corporation (Beijing, China). The mice were allowed to acclimate to surroundings at a temperature range of 22 to 24 °C for one week and maintained under a 12-hour light and dark cycle for adaptation. All animal procedures were approved by the Fudan University Institutional Animal Care and Use Committee and conducted in accordance with guidelines from the Department of Laboratory Animal Science at Fudan University. Seven-week-old mice were treated daily with 62.5 mg IMQ (5%) (Sichuan Med-shine Pharmaceutical, H20030128) on a shaved back area (3 × 2 cm) for six consecutive days to induce the psoriasis-like mouse model (n = 6), the model with agomir-125a-5p treatment (n = 6) and the model with negative control agomir (agomir-NC) treatment (n = 6). Control mice (n = 3) were treated similarly with the same amount of Vaseline.

### Intradermal injection of agomir-125a-5p

miR-125a-5p agomir and its negative controls were dissolved and mixed according to the manufacturer’s instructions (RiboBio, China). On day 0, 1, 2, 3, and 4, a single dose of agomir-125a-5p (4 nmol/day), and its corresponding controls were intradermally injected into the back skin of mice before the application of IMQ. On the sixth day, all mice were sacrificed. The oligonucleotide sequences of agomir-125a-5p were as follows: 5′-UCCCUGAGACCCUUUAACCUGUGA-3′.

### Modified PASI scoring

An improved PASI scoring system was used to assess the severity of back skin inflammation in the experimental mice [20]. Erythema, scaling, and thickness were scored on a scale of 0 to 4 as follows: 0 for none, 1 for mild, 2 for moderate, 3 for severe, and 4 for very severe. The total scores ranged from 0 to 12 for each mouse. Two independent investigators blinded to the experimental groups of mice performed the scoring simultaneously.

### Histological analysis

On the sixth day, 8 h after the last injection of agomir-125a-5p, the mice were euthanized. Mouse skin was harvested, fixed in 4% paraformaldehyde (G1101, Servicebio, China) in PBS, dehydrated, and embedded at the optimal temperature. Hematoxylin and Eosin (H&E) staining was performed following standard protocols. Peripheral blood and spleens were also collected.

### Cell transfection and cell culture

CD4^+^ T lymphocytes from mice spleen were plated in 6-well plates at a density of 2 × 10^6^ cells/well using Lipofectamine 3000, following the manufacturer’s protocols. The cells were co-cultured with either mimic Negative Controls (mimic NCs) or miR-125a-5p mimics for 48 h. After transfection, cells were collected for flow cytometry and quantitative real-time PCR (qRT-PCR). The miR-125a-5p mimics, mimic NCs were synthesized by Guangzhou RiboBio Co., Ltd. The oligonucleotide sequences of miR-125a-5p were as follows: 5′-UCCCUGAGACCCUUUAACCUGUGA-3′. The oligonucleotide sequences of mimic NCs were based on the sequences of miRNA in Caenorhabditis elegans (cel-miR-67).

### Flow cytometry

Peripheral blood mononuclear cells (PBMCs) were isolated from blood samples of psoriatic patients and healthy donors by Ficoll-Paque gradient centrifugation (17144003, Cytiva). Human CD4^+^ T cells were isolated using a CD4^+^ T cell isolation kit (Cat#480,009, BioLegend) following the manufacturer’s protocol. Peripheral blood was obtained from experimental mice, and mouse CD4^+^ T cells were isolated using a CD4^+^ T cell isolation kit (Cat#19,852, Stem cell) following the manufacturer’s protocol. The human and mouse CD4^+^ T cell purity was confirmed to be above 95% by flow cytometric analysis. Flow cytometry was performed using FACS Calibur (BD Biosciences) and Cytoflex (Beckman) devices. Data were analyzed using FlowJo software (Tree Star, Ashland, OR). Th17 cells were defined as CD4^+^ IL17^+^ cells, and Tregs were defined as CD4^+^CD25^+^ Foxp3^+^ cells.

The following antibodies were used: FITC rat anti-human CD4 (Biolegend), APC mouse anti-human CD4 (Biolegend), PE/Cyanine5 mouse anti-human CD25 (Biolegend), PE mouse anti-human IL-17 A (eBioscience), FITC rat anti-mouse CD4 (Biolegend), PE rat anti-mouse CD3 (Biolegend), PE rat anti-mouse IL-17 A (Biolegend), and APC rat anti-mouse CD25 (Biolegend).

### RNA isolation and quantitative real-time PCR

Total RNA was extracted from CD4^+^ T cells in peripheral blood samples of psoriatic patients, healthy donors, and normal mice using TRIzol Reagent (Ambion, Thermo Fisher Scientific). RNA quality was assessed using a NanoDrop spectrophotometer (ND-ONE-W, Thermo Fisher Scientific). mRNA was reverse transcribed using the ReverTra Ace qPCR RT Kit (TOYOBO, Japan), and miRNA was reverse transcribed using the miScript II RT Kit (QIAGEN) according to the manufacturer’s instructions. qPCR was performed using the AceQ qPCR SYBR Green Master Mix (Vazyme) on a LightCycler 480 II thermocycler (Roche). PCR was performed using the miScript SYBR Green PCR kit (QIAGEN) on an S1000 PCR machine (Bio-Rad). Primers for miR-125a-5p and U6 were purchased from RiboBio Co., and other relevant primers are listed in Additional file 1: Table S2.

### Statistical analysis

The data are presented as mean ± standard deviation. Data analysis was conducted using GraphPad Prism 5 (GraphPad Software, San Diego, CA) with Student’s t-test and the Mann-Whitney test. A p-value less than 0.05 was considered statistically significant.

## Results

### Downregulated expression of miR-125a-5p in CD4^+^T cells of peripheral blood in psoriatic patients and its relationship with Th17 /Treg balance and psoriasis severity

**Fig. 1:**
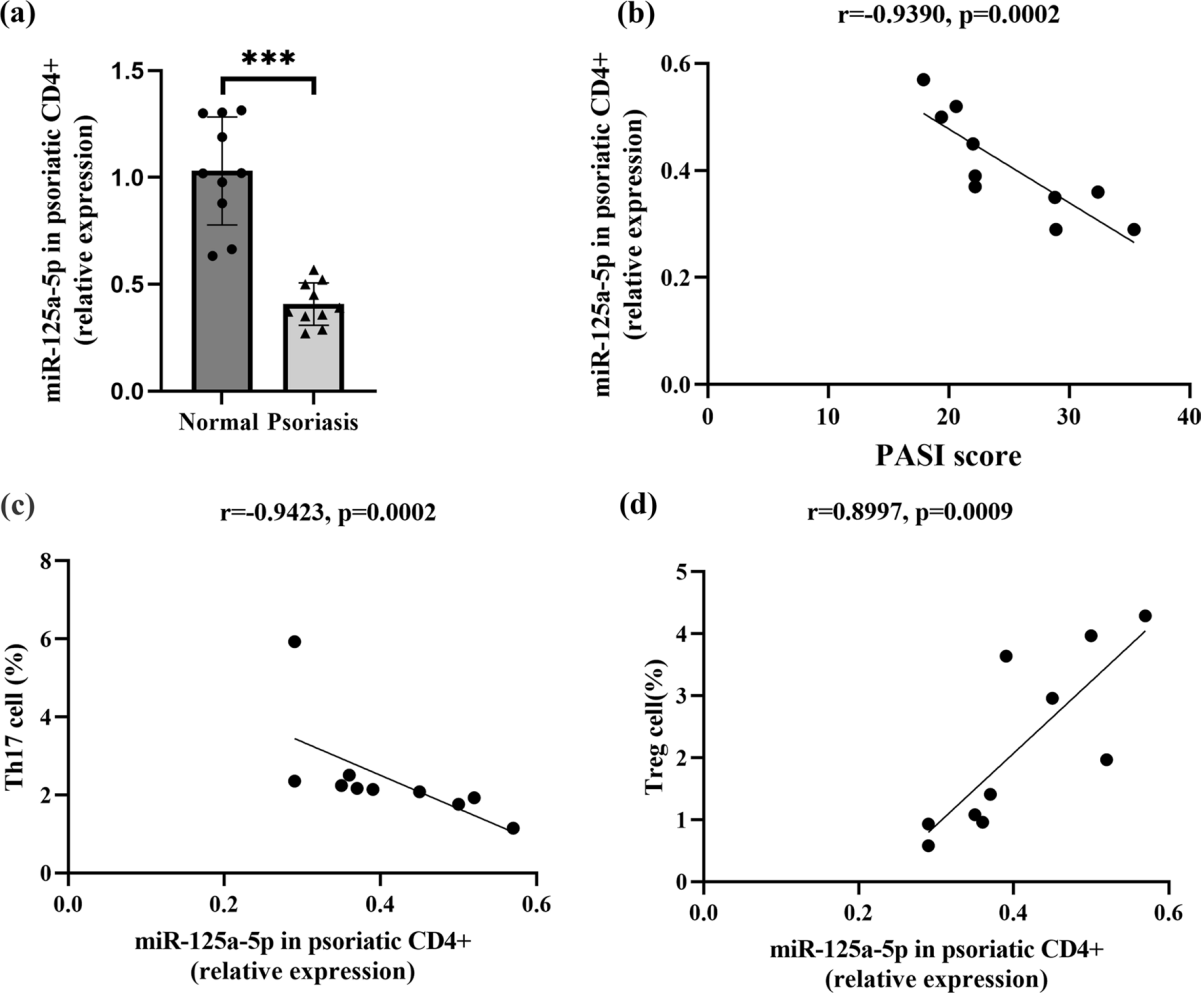
miR-125a-5p expression is reduced in CD4^+^T cells, linked to Th17/Treg balance and psoriasis severity. **a** miR125a-5p expression in peripheral CD4^+^ T cells of psoriatic patients and normal healthy controls were analyzed by qRT-PCR (n = 10 per group, p < 0.001). **b** Negative correlation of human miR-125a-5p expression in psoriatic CD4^+^ T cells with PASI scores (n = 10, p < 0.001). **c** Negative correlation of miR-125a-5p expression in psoriatic CD4^+^ T cells with the proportion of Th17 cells in peripheral CD4^+^ T cells of psoriasis patients (n = 10, p < 0.001). **d** Positive correlation of miR-125a-5p expression in psoriatic CD4^+^ T cells with the proportion of Treg cells in peripheral CD4^+^ T cells of psoriasis patients (n = 10, p < 0.001). Data are representative of three independent experiments (mean ± SEM). *P < 0.05, **P < 0.01, ***P < 0.001. Two-tailed unpaired Student’s t-test **a**, Spearman’s r test **b**, **c** and **d**. *miR* microRNA; *qRT-PCR* quantitative real-time PCR; *PASI* psoriasis area and severity index; Th17, T helper cell 17; *Treg* regulatory T cel

We measured the expression of miR-125a-5p in peripheral blood CD4^+^ T cells of psoriatic patients and found that it was significantly decreased compared to healthy controls (Fig. 1a). Notably, we observed a negative correlation between miR-125a-5p expression in CD4^+^ T cells and the PASI scores of psoriasis patients (Fig. 1b). CD4^+^ T helper (Th) cells play a crucial role in psoriasis pathogenesis, and the imbalance of Th subsets, including Th1/Th2 and Th17/Treg subsets, contributes to the immunopathology of psoriasis [21, 22]. We sorted and verified CD4^+^ T cells from PBMCs of healthy individuals and psoriatic patients. We found that the percentage of Th17 cells in CD4^+^ T cells was significantly higher in psoriatic patients compared to healthy controls (Additional file 1: Figure S1a). However, the percentage of Tregs in CD4^+^ T cells was significantly lower in psoriatic patients compared to healthy subjects (Additional file 1: Figure S1b). The dysregulation of the Th17/Treg balance in psoriasis patients is consistent with previous studies. Furthermore, through correlation analysis, we detected a negative correlation between miR-125a-5p expression and the percentage of Th17 cells in human CD4^+^ T cells (Fig. 1c), as well as a positive correlation between miR-125a-5p expression and the percentage of Tregs in human CD4^+^ T cells (Fig. 1d). In summary, these data demonstrate that the downregulation of miR-125a-5p expression in peripheral CD4^+^ T cells may be associated with the imbalanced proportion of Th17/Treg cells in psoriatic patients.

### Upregulated expression of miR-125a-5p putative targets ETS-1, IFN-γ, and STAT3 in peripheral CD4^+^T cells of psoriatic patients

**Fig. 2:**
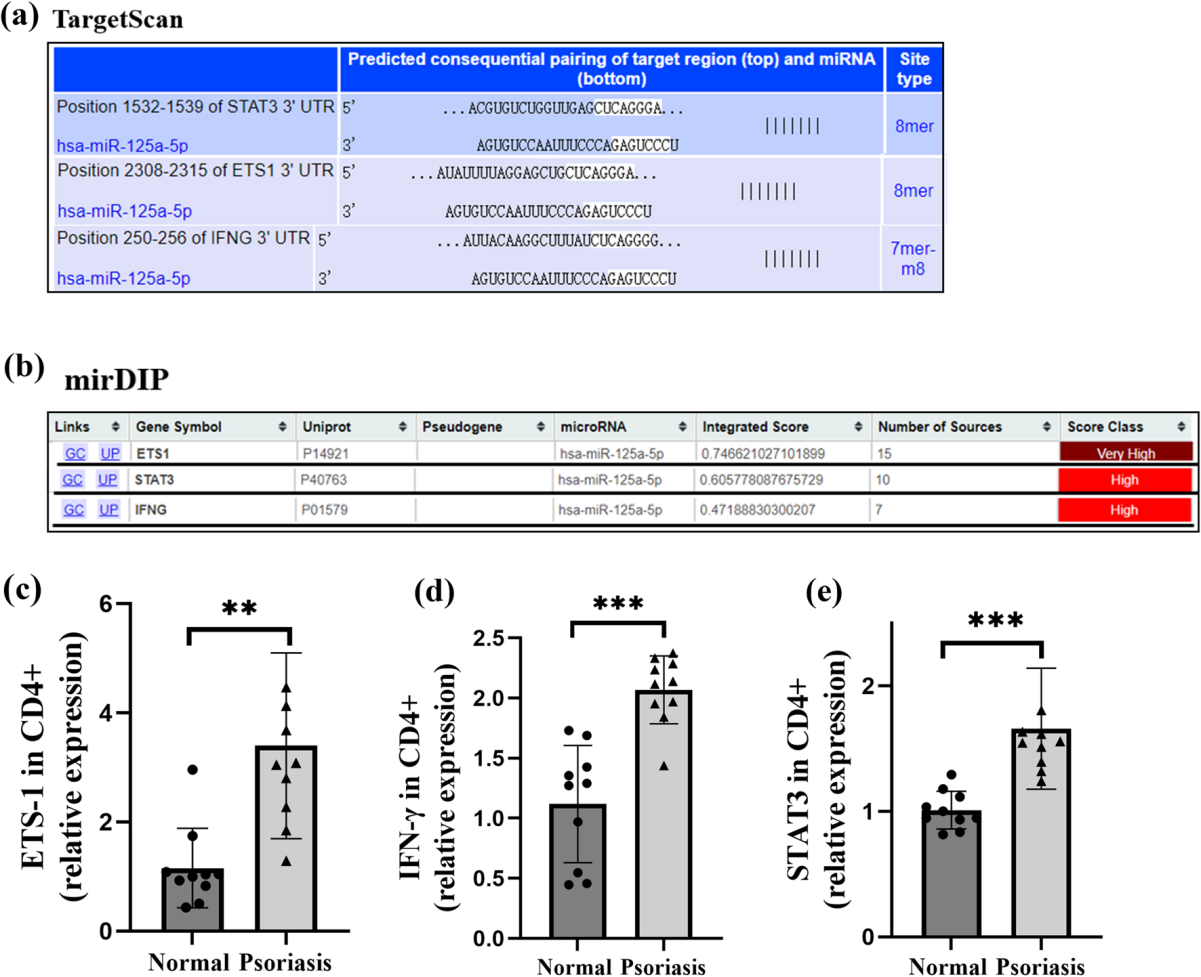
Upregulated expression of miR-125a-5p targets ETS-1, IFN-γ, and STAT3 in psoriatic CD4^+^T cells. **a**,** b** ETS-1, IFN-γ, and STAT3 were predicted to be targeted by miR-125a-5p using TargetScan (**a**) and mirDIP (**b**). **c** ETS-1 expression in peripheral CD4^+^ T cells of psoriasis patients and normal healthy controls was analyzed by qRT-PCR (n = 10 per group, p < 0.01). **d** IFN-γ expression in peripheral CD4^+^ T cells of psoriasis patients and normal healthy controls was analyzed by qRT-PCR (n = 10 per group, p < 0.001). **e** STAT3 expression in peripheral CD4^+^ T cells of psoriasis patients and normal healthy controls was analyzed by qRT-PCR (n = 10 per group, p < 0.001). Data are representative of three independent experiments (mean ± SEM). *P < 0.05, **P < 0.01, ***P < 0.001. Two-tailed unpaired Student’s t-test (**c**,** d** and **e**). *miR* microRNA; *STAT3* Signal transducer and activator of transcription 3; *ETS-1* E26 transformation specific-1; *IFN-γ* Interferon-γ; *qRT-PCR* quantitative real-time PCR

According to the target prediction by two known databases for target prediction, TargetScan (http://www.targetscan.org/) and mirDIP (http://ophid.utoronto.ca/mirDIP/) [23], ETS-1, IFN-γ, and STAT3 are the most likely targets of miR-125a-5p (Fig. 2a and b). Among these, ETS-1 [17] and IFN-γ [22] have been reported to be involved in Th1 cell differentiation, while STAT3 [24] has been reported to participate in Th17 cell differentiation. We used qRT-PCR analysis to confirm that mRNA levels of ETS-1, IFN-γ, and STAT3 were significantly upregulated in peripheral blood CD4^+^ T cells of psoriatic patients compared to healthy donors (Fig. 2c–e).

### miR-125a-5p mimic drives differentiation of murine splenic CD4^+^ T cells to Tregs

**Fig. 3:**
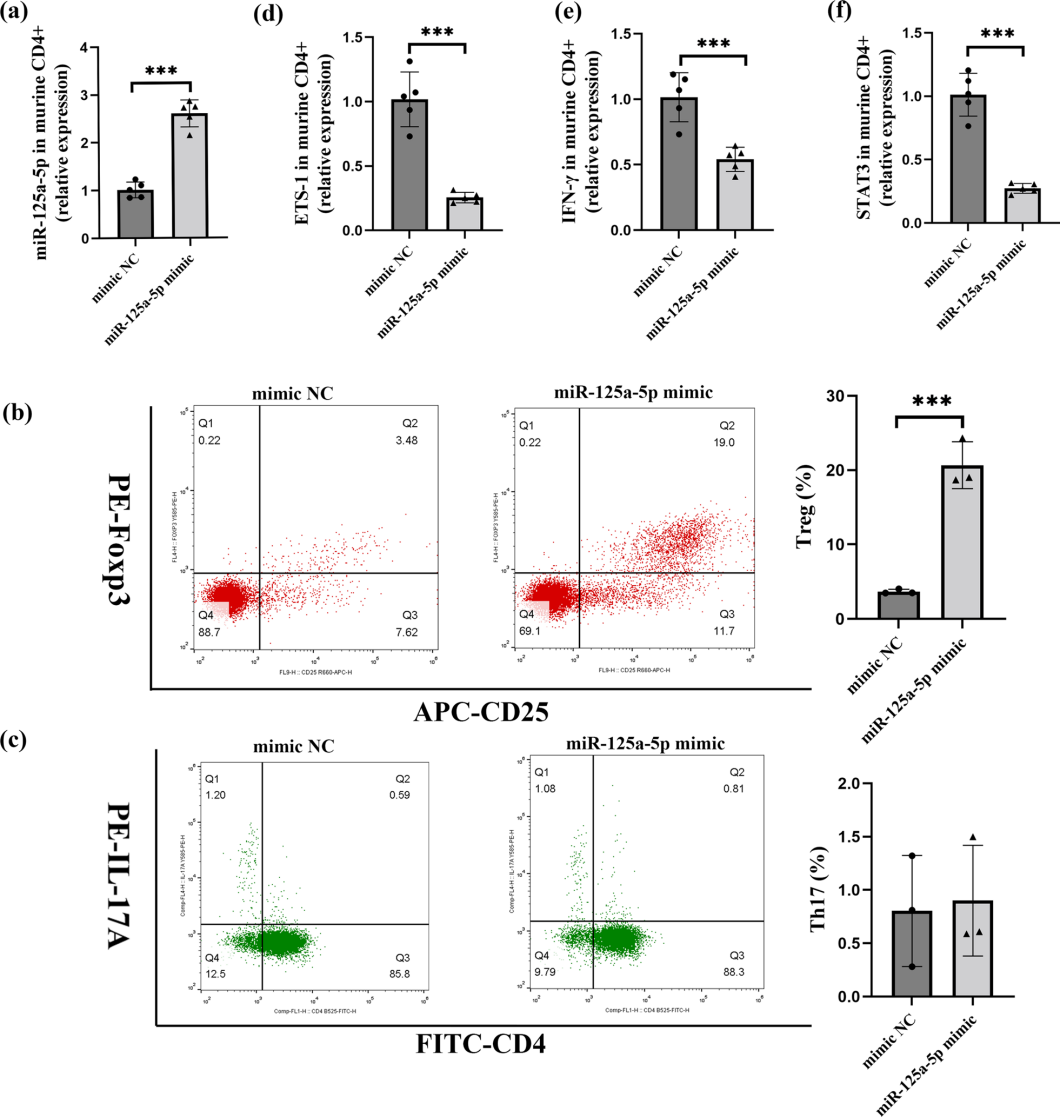
miR-125a-5p mimic drives differentiation of murine splenic CD4^+^T cells to Tregs. **a** miR-125a-5p expression in normal murine splenic CD4^+^ T cells transfected with miR-125a-5p mimic or mimic NC for 48 h by qRT-PCR (n = 5 per group, p < 0.001). **b** The proportion of CD25^+^ Foxp3^+^ T cells in normal murine splenic CD4^+^ T cells transfected with miR125a-5p mimic or mimic NC for 48 h by flow cytometry (n = 3 per group, p < 0.001). **c** The proportion of CD4^+^ IL17 ^+^ T cells in normal murine splenic CD4^+^ T cells transfected with miR125a-5p mimic or mimic NC for 48 h by flow cytometry (n = 3 per group, not significant). **d** ETS-1 expression in normal murine splenic CD4^+^ T cells transfected with miR125a-5p mimic or mimic NC for 48 h by qRT-PCR (n = 5 per group, p < 0.001). **e** IFN-γ expression in normal murine splenic CD4^+^ T cells transfected with miR125a-5p mimic or mimic NC for 48 h by qRT-PCR (n = 5 per group, p < 0.001). **f **STAT3 expression in normal murine splenic CD4^+^ T cells transfected with miR125a-5p mimic or mimic NC for 48 h by qRT-PCR (n = 5 per group, p < 0.001). Data are representative of three independent experiments (mean ± SEM). *P < 0.05, **P < 0.01, ***P < 0.001. Two-tailed unpaired Student’s t-test **a**, **b**, **c**,** d**,** e** and **f** *miR* microRNA; *Tregs* regulatory T cells; *mimic NC* mimic Negative Control; *qRT-PCR* quantitative real-time PCR; *Foxp3* Fork head transcription factor3; *IL* interleukin; *STAT3* Signal transducer and activator of transcription 3; *ETS-1* E26 transformation specific-1; *IFN-γ* Interferon-γ

To directly assess whether miR-125a-5p modulates Th cell differentiation, we sorted and confirmed CD4^+^ T cells from murine splenic lymphocytes (not shown). Next, we transfected murine CD4^+^ T cells with a miR-125a-5p mimic (Fig. 3a) and measured the proportions of Th17 cells and Tregs. Interestingly, the results showed that increased miR-125a-5p expression promoted the differentiation of murine CD4^+^ T cells to Tregs (Fig. 3b) but had no significant effect on the differentiation of Th17 cells (Fig. 3c). We then evaluated whether overexpression of miR-125a-5p influences the endogenous expression of ETS-1, IFN-γ, and STAT3 in murine splenic CD4^+^ T cells. As expected, overexpression of miR-125a-5p significantly decreased the mRNA levels of ETS-1, IFN-γ, and STAT3 in splenic CD4^+^ T cells of mice transfected with miR-125a-5p mimic compared to the mimic NC group (Fig. 3d–f).

### Intradermal administration of agomir-125a-5p ameliorates the phenotype of IMQ-induced psoriasis-like inflammation

**Fig. 4:**
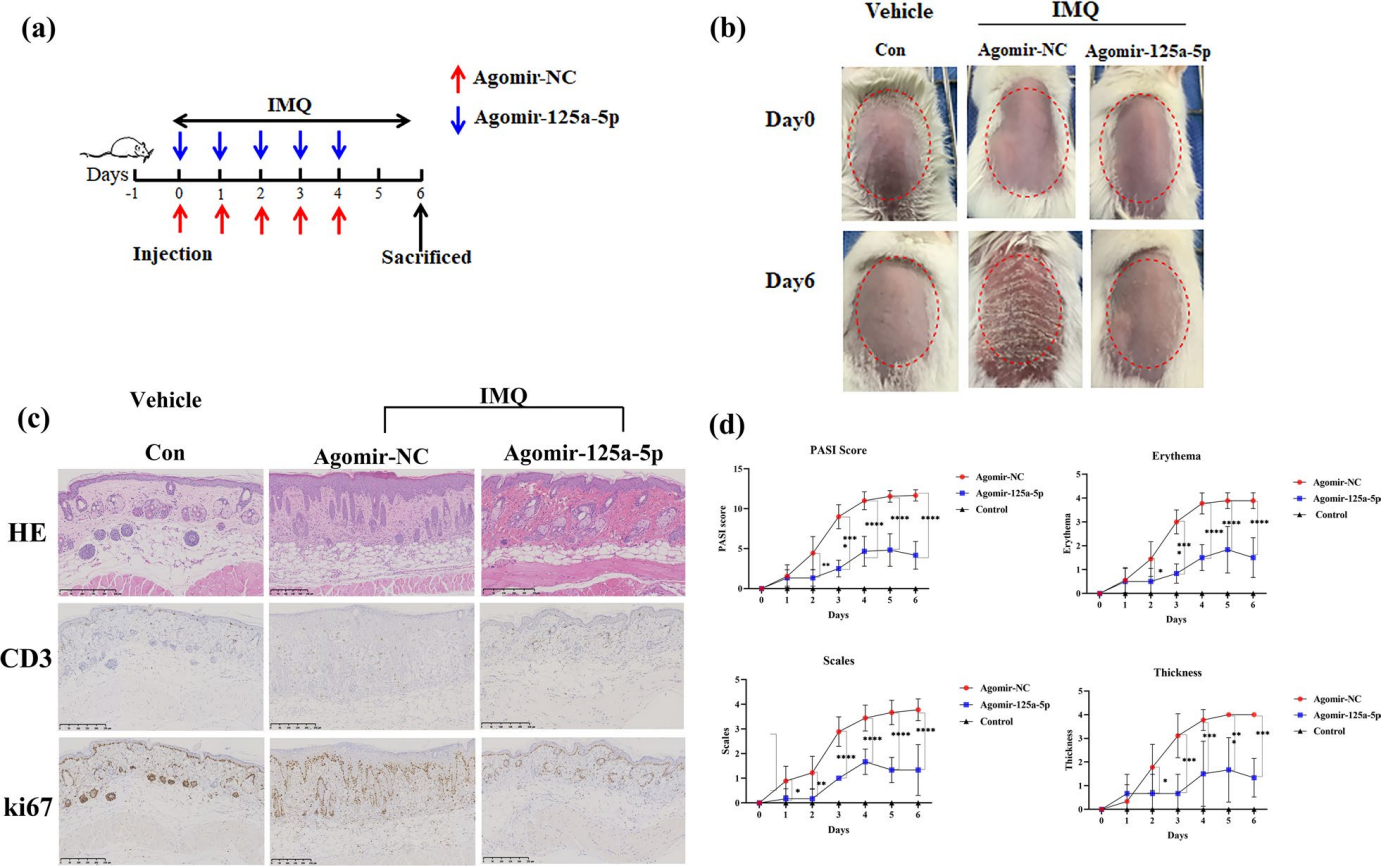
Intradermal administration of agomir-125a-5p ameliorates the pathological phenotype of IMQ-induced psoriasis-like mice. **a** Schematic diagram of the intradermal administration of agomir-125a-5p (4 nmol/day) and agomir-NC (4 nmol/day) on days 0, 1, 2, 3, and 4 after application of IMQ in mice (BALB/c). The mice in each group were sacrificed on day 6. **b** Phenotypic presentation of back skin lesions of each group on day 0 and day 6. **c** Representative H&E staining of back lesions of each group on day 6 (×200) (**upper panels**). Representative immunohistochemistry sections of ki67 and CD3 staining (**brown**) of the back skin lesions (**middle and bottom panels**). Scale bar: 50 μm. **d** Improved PASI scoring of skin lesions, including erythema, scaling, thickness, and total PASI scores. As in thickness, there was no standard deviation on day 5 and day 6 since the scores remained at 4. Data are representative of at least two independent experiments (mean ± SEM). *P < 0.05, **P < 0.01, ***P < 0.001, ****P < 0.0001. Two-tailed unpaired Student’s t-test (**d**). miR, microRNA; agomir-NC, negative control agomir; Con, control; IMQ, imiquimod; H&E staining, Hematoxylin and Eosin staining; PASI, Psoriasis Area and Severity Index

Our findings reveal that miR-125a-5p plays a pivotal role in regulating Th17 cells and Tregs, which are fundamental cells in the key controlling pathway of psoriasis pathogenesis. Therefore, we investigated the potential therapeutic and application values of miR-125a-5p. We constructed an IMQ-induced psoriasis mouse model and injected agomir-125a-5p or agomir-NC intradermally 6 h after applying IMQ for consecutive 6 days to validate the protective efficacy of miR-125a-5p in the IMQ-induced psoriasis-like mouse model (Fig. 4a). As expected, compared to mice in the agomir-NC group, there was a significant improvement in both clinical and pathological characteristics of mice on day 6 after five days of injections of agomir-125a-5p (Fig. 4b and c), along with a markedly decreased disease severity on erythema, scaling, and thickness (Fig. 4d).

### Agomir-125a-5p downregulates the proportion of Th17 cells in peripheral blood of IMQ-induced psoriasis-like mice

**Fig. 5:**
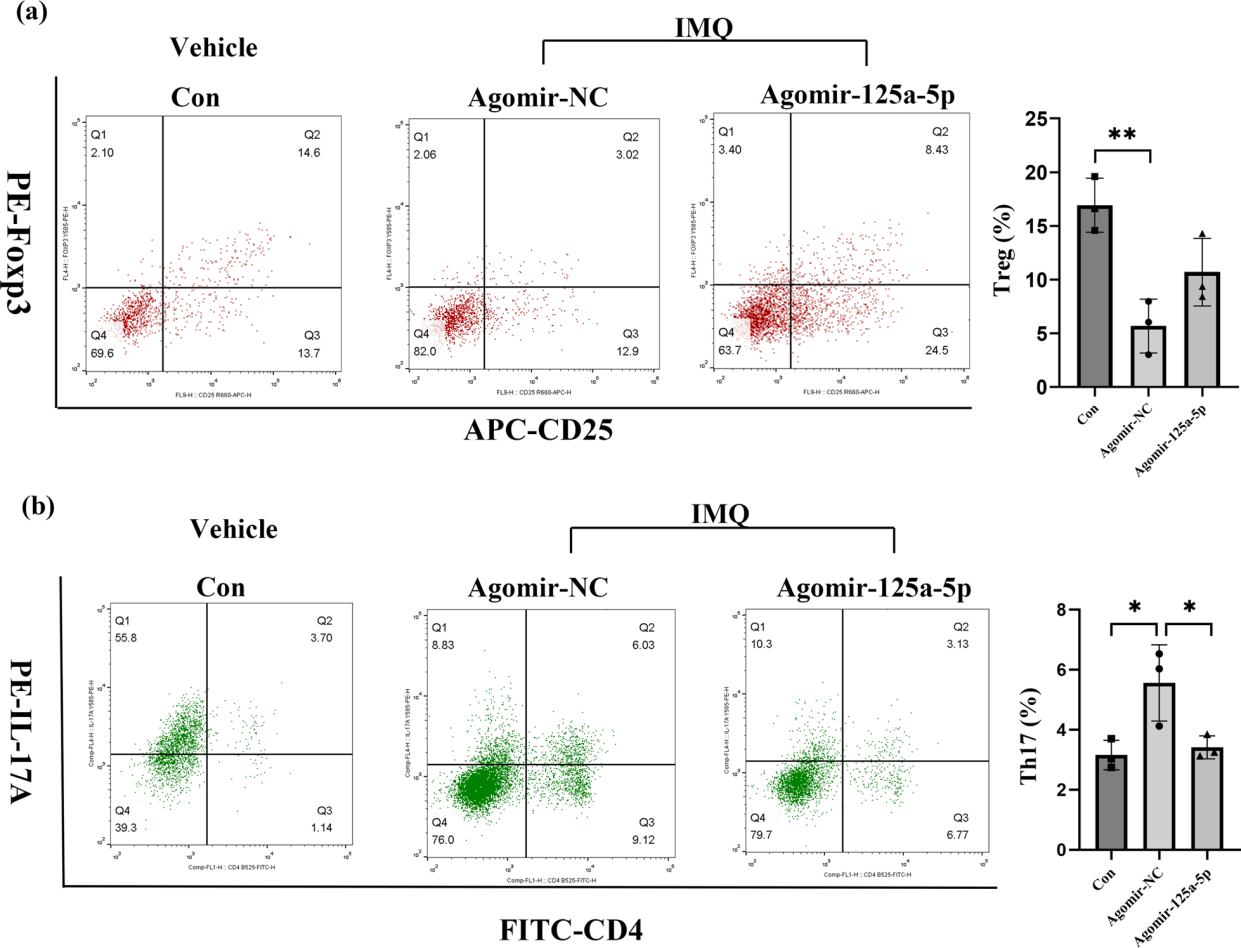
Agomir-125a-5p downregulates the proportion of Th17 cells in peripheral blood of IMQ-induced psoriasis-like mice. The proportion of CD25^+^ Foxp3^+^ T cells in peripheral blood of mice from each group by flow cytometry (n = 3, p < 0.01). The proportion of CD4^+^ IL17 ^+^ T cells in peripheral blood of mice from each group by flow cytometry (n = 3, p < 0.05). Data are representative of at least two independent experiments (mean ± SEM). *P < 0.05, **P < 0.01. Two-tailed unpaired Student’s t-test (**a**,** b**). *miR* microRNA; *agomir-NC *negative control agomir; *Con* control; *IMQ* imiquimod; *Foxp3* Fork head transcription factor3; *IL* interleukin

 To investigate the impact of miR-125a-5p in regulating Th17/Treg cells in IMQ-induced psoriasis-like inflammation in vivo, we measured the proportions of Th17 cells and Tregs in CD4^+^ T cells of mice in the agomir-125a-5p group. We found a relatively increased proportion of CD4^+^ CD25^+^ Foxp3^+^ Tregs (Fig. 5a) and a significant downregulation of the proportion of CD4^+^ IL17^+^ Th17 cells CD4^+^ lymphocytes (Fig. 5b) in mice in the agomir-125a-5p group compared to mice in the agomir-NC group.

## Discussion

Our study found that miR-125a-5p was significantly downregulated in the peripheral blood CD4^+^ T cells of psoriatic patients and negatively correlated with the PASI score. These results are also consistent with Su’s study, which found that miR-125a expression was downregulated in psoriatic skin tissues and negatively associated with the PASI score [25]. Su et al. reported that overexpression of miR-125a inhibited the proliferation of HaCaT cells and promoted apoptosis, while miR-125a knockdown enhanced the proliferation of HaCaT cells and inhibited apoptosis through the IL23R/JAK2/STAT3 pathway in HaCaT cells. However, they only analyzed the expression of miR-125a in skin lesions and did not investigate its specific localization.

We believe that immune cells play a more significant role in the function of miR-125a and therefore sought to investigate the role of miR-125a in distinct T cell subpopulations of psoriatic patients.

A previous study showed that miR-125a-5p knockout mice had a significantly reduced proportion of Forkhead box P3^+^(Foxp3^+^) Tregs compared to wild-type (WT) mice, and T cells lacking miR-125a exhibited a reduced ability to differentiate into Foxp3^+^ Treg cells compared with WT T cells [13]. Furthermore, miR-125a-5p was identified as a commonly downregulated miRNA in CD4^+^ T cells of various autoimmune diseases, including Crohn’s disease [2, 13], inflammatory bowel diseases [15], and SLE [14], indicating its potential negative regulatory role in inflammation. Therefore, we aim to investigate the role of miR-125a-5p in CD4^+^ T cells of psoriatic patients.

Our results showed that miR-125a-5p expression in peripheral CD4^+^ T cells was positively associated with the proportion of Treg cells and negatively correlated with the proportion of Th17 cells in psoriatic patients. In vitro experiments demonstrated that miR-125a-5p mimics promoted the differentiation of murine splenic CD4^+^ T cells into Tregs, while no significant impact was observed on the differentiation of Th17 cells, which was consistent with a previous study that found defective Treg differentiation in miR-125a deficient T cells.

We further intradermally injected agomir-125a-5p into the back skin of IMQ-induced psoriasis-like mice and confirmed the function of miR-125a-5p in alleviating psoriasis-like inflammation, downregulating the proportion of Th17 cells and upregulating the proportion of Tregs compared to mice in the agomir-NC group. These findings establish miR-125a-5p as a crucial regulator in controlling the differentiation of CD4^+^ T cells into Tregs, thereby alleviating the inflammatory response.

In this study, we observed upregulated levels of ETS-1 and IFN-γ mRNA in peripheral CD4^+^ T cells of psoriatic patients and found that miR-125a-5p mimic downregulated the expression of ETS-1 and IFN-γ mRNA in murine splenic CD4^+^ T cells, suggesting a potential negative regulatory effect of miR-125a-5p on ETS-1 and IFN-γ. Previous studies utilizing RNA-sequencing have shown increased expression of ETS-1 in miR-125a^−/−^ CD4^+^ T cells, and dual-luciferase assays have confirmed this target [15, 26]. ETS-1 is a transcription factor belonging to the ETS family, which is mainly expressed in immune cells such as T cells and plays a role in T cell differentiation and activation [27, 28]. Knockdown of ETS-1 in CD4^+^ T cells inhibits the differentiation of Th1 cells and subsequently downregulates the levels of IFN-γ [15]. Th1 cells and their product, IFN-γ, are proinflammatory factors involved in psoriasis pathogenesis [29, 30]. Research has suggested that ETS-1 can boost IFN-γ production in Th1 cells by targeting the IFN-γ promoter and promote Th1 cells differentiation by upregulating the T-bet gene [17]. Thus, the decreased miR-125a-5p in CD4^+^ T cells of psoriatic patients may upregulate the transcripts levels of its downstream targets, ETS-1, and IFN-γ.

Our results showed that overexpression of miR-125a-5p in murine splenic CD4^+^ T cells drove their differentiation into Tregs. It is reported that miR-125a-5p is highly expressed in Tregs and stabilizes the commitment and immunoregulatory capacity of Tregs by inhibiting effector T lineage factors [12, 13]. In the absence of miR-125a-5p, Tregs display increased expression of effector T genes, including T-bet and IFN-γ, and decreased expression of Foxp3, the Treg-specifying gene [13]. T-bet and IFN-γ are Th1 polarizers that can inhibit the differentiation of Foxp3-expressing Treg cells from naïve CD4^+^ T cells [31–33]. Therefore, we speculate that the reduction of miR-125a-5p in psoriatic CD4^+^ T cells might downregulate the suppressive effect of Tregs to Th1 cells indirectly through targeting ETS-1 and IFN-γ.

We also discovered that the expression of STAT3 mRNA was upregulated in psoriatic CD4^+^ T cells, and miR-125a-5p mimics downregulated the expression of STAT3 mRNA in murine splenic CD4^+^ T cells, suggesting a suppressive impact of miR-125a-5p on STAT3. RNA-sequencing showed increased STAT3 expression in miR-125a^−/−^ CD4^+^ T cells, which was verified as a target of miR-125a by luciferase assay [15, 34, 35]. STAT3 plays a critical role in differentiating both Th17 cells and Tregs from naïve CD4^+^ precursors [36], and the mechanism by which miR-125a-5p regulates the immune response of CD4^+^ T cells in psoriasis pathogenesis may also be associated with STAT3.

STAT3 facilitates Th17 cell differentiation by mediating cytokine responses to IL-6 and IL-21, which initiate Th17 cell differentiation [24, 37]. Additionally, it promotes the expression of molecules, including RORγ, RORα, IL-23R, and IL-17, that are essential for Th17 cell development [38, 39]. Furthermore, STAT3 signalling through IL-23R in the presence of IL-23 further enhances Th17 cell differentiation, while secreted IL-17 performs important effector functions of this subset [22, 40]. By contrast, STAT3 inhibits the generation of Tregs and suppresses the expression of Foxp3 in mature Tregs [41, 42]. Studies have shown that inhibition of STAT3 signalling enhances the differentiation and function of Treg cells by upregulating Foxp3 [43, 44]. Tregs from the peripheral blood of psoriatic patients have been reported to show reduced suppressive function together with STAT3 phosphorylation [45, 46]. Taken together, the downregulation of miR-125a-5p in psoriatic CD4^+^ T cells may reduce the inhibitory effect of Treg cells on Th17 cells by targeting STAT3.

Limitations of this study using miR-125a-5p include: First, the relatively small sample size (30 psoriasis patients and 30 sex-matched healthy donors), that could slightly reduce statistical capacity. Second, the lack of a further disease control group. Third, we speculated that miR-125a-5p in psoriatic CD4 + T cells might reduce the inhibitor role of Tregs on Th1 cells indirectly through targeting ETS-1 and IFN-γ. Further experiments should be done to confirm the regulatory effect of miR-125a-5p on Tregs to Th1 cells. Although we clarified that miR-125a-5p restored the suppressive effect of Tregs on Th1 and Th17 cells, we believe that it might be only a tip of iceberg. For further understanding the role of miR-125a-5p in psoriasis pathogenesis, more experiments are needed to determine its effects on other immune cells, and skin microbes.

## Conclusions

In conclusion, the present study provides evidence that miR-125a-5p may have a therapeutic potential in psoriasis by restoring the suppressive function of Tregs on Th17 cells through targeting STAT3, and on Th1 cells indirectly through targeting ETS-1 and IFN-γ.

### Supplementary Information

**Additional file 1: Table S1.** Patient characteristics. **Table S2.** Primer sequences for reverse-transcription quantitative polymerase chain reaction in humans and mice. **Figure S1.** The upregulated Th17 cells and downregulated Tregs in peripheral blood of psoriatic patients. **a** The proportion of CD4+IL17 + T cells in peripheral blood of psoriatic patients and healthy controls by flow cytometry (n=10 per group, p<0.001). **b** The proportion of CD25+Foxp3+ T cells in peripheral blood of psoriatic patients by flow cytometry (n=10 per group, p<0.01). Data are representative of three independent experiments (mean±SEM). *P<0.05, **P<0.01, ***P<0.001. Two-tailed unpaired Student’s t-test (**a**, **b**). Th17, T helper cell 17; Treg, regulatory T cell; IL, interleukin; Foxp3, Forkhead transcription factor3.

## Data Availability

Reasonable requests for data will be made available for review.

## Abbreviations

ETS-1: E26 transformation specific-1
Foxp3+: Forkhead box p3+
IFN-γ: Interferon-γ
IMQ: Imiquimod
miR: MicroRNA
mRNA: Messenger RNA
NC: Negative control
PASI: Psoriasis Area and Severity Index
PBMCs: Peripheral blood mononuclear cells
qRT-PCR: Quantitative real-time PCR
SLE: Systemic lupus erythematosus
STAT3: Signal transducer and activator of transcription 3
Th: Helper T
Tregs: Regulatory T cells
WT: Wild-type
3′ UTRs: 3′ untranslated regions
