# Supplementary material for: MicroRNA-125a-5p regulates the effect of Tregs on Th1 and Th17 through targeting ETS-1/STAT3 in psoriasis
Source: J Transl Med. 2023 Sep 29;21:678. doi: 10.1186/s12967-023-04427-6 (PMC10543306; doi:10.1186/s12967-023-04427-6)

**Supplemental data**

**Table S1**. **Patient characteristics.**

|  | **Psoriasis** | **Healthy controls** |
| --- | --- | --- |
| **n** | **30** | **30** |
| **Mean age, years(range)** | **45(22-64)** | **41(25-58)** |
| **Sex, male/female** | **18/12** | **20/10** |
| **Mean disease duration, years(range)** | **16(1-30)** | **NA** |
| **Mean PASI score** | **18.6±4.5** | **NA** |

**Table S2**. **Primer sequences for reverse-transcription quantitative polymerase chain reaction in humans and mice.**

| Target | Sequence (5′-3′) |
| --- | --- |
| Human miR-125a-5p | F: CCTGAGACCCTTTAACC |
|  | R: CCAATTTCCCAGAGTCC |
| Human-U6 | F: TTCGTGAAGCGTTCCATATTTT |
|  | R: TTTTATACCTTGCGAAGTGCTT |
| Human-STAT3 | F: ATCACGCCTTCTACAGACTGC |
|  | R: CATCCTGGAGATTCTCTACCACT |
| Human-ETS-1 | F: GACAGCTTCGACTCAGAGGA |
|  | R: ACTGCCATAGCTGGATTGGT |
| Human-IFN-γ | F: TGAATGTCCAACGCAAAGCA |
|  | R: TCGACCTCGAAACAGCATCT |
| Human-β-actin | F: CTGGAACGGTGAAGGTGACA |
|  | R: CGGCCACATTGTGAACTTTG |
| Mouse-miR-125a-5p | F: CCCTGAGACCCTTTAACC |
|  | R: CCAATTTCCCAGAGTCCC |
| Mouse-U6 | F: GACTACAGGCATTGTGGAGACC |
|  | R: CCAGAGGTGTTACGGACATCAG |
| Mouse-STAT3 | F: AGGAGTCTAACAACGGCAGCCT |
|  | R: GTGGTACACCTCAGTCTCGAAG |
| Mouse-ETS-1 | F: CCAGAATCCTGTTACACCTCGG |
|  | R: CAGCGTCTGATAGGACTCTGTG |
| Mouse-IFN-γ | F: TGAACGCTACACACTGCATCTTGG |
|  | R: CGACTCCTTTTCCGCTTCCTGAG |
| Mouse-β-actin | F: CATTGCTGACAGGATGCAGAAGG |
|  | R: TGCTGGAAGGTGGACAGTGAGG |

Note: miR-125a-5p, microRNA-125a-5p; STAT3, signal transducers and activators of transcription 3; ETS-1, E26 transformation specific-1; IFN-γ, Interferon-γ; F, forward; R, reverse.

**Figure S1.** **The upregulated Th17 cells and downregulated Tregs in peripheral blood of psoriatic patients.**

1. The proportion of CD4^+^ IL17 ^+^ T cells in peripheral blood of psoriatic patients and healthy controls by flow cytometry (n=10 per group, p<0.001)
2. The proportion of CD25^+^ Foxp3^+^ T cells in peripheral blood of psoriatic patients by flow cytometry (n=10 per group, p<0.01).

Data are representative of three independent experiments (mean±SEM). *P<0.05, **P<0.01, ***P<0.001. Two-tailed unpaired Student’s t-test (**a-b**).

Th17, T helper cell 17; Treg, regulatory T cell; IL, interleukin; Foxp3, Forkhead transcription factor3.


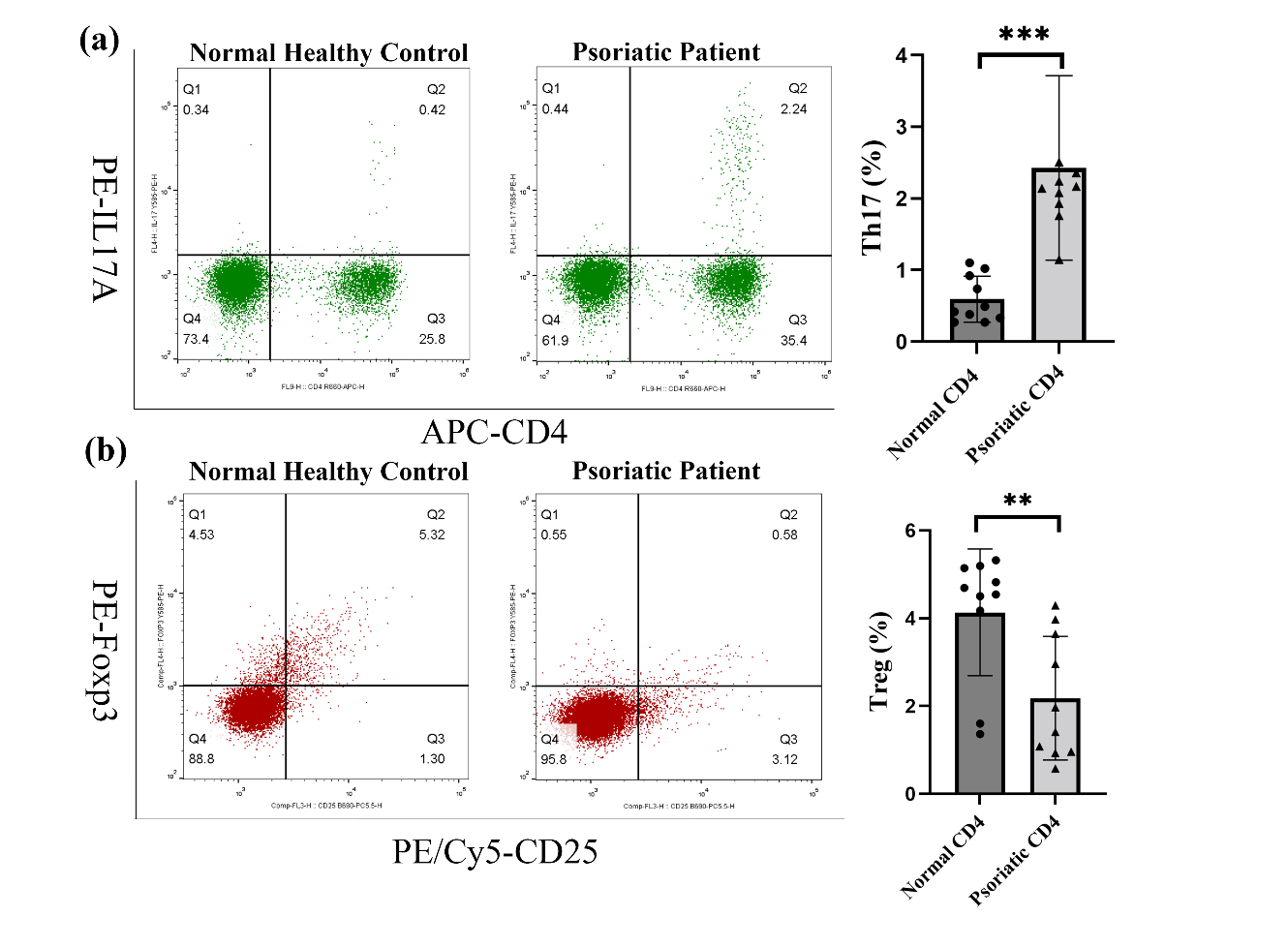

Supplement: Supplementary file 1 — Additional file 1: Table S1. Patient characteristics. Table S2. Primer sequences for reverse-transcription quantitative polymerase chain reaction in humans and mice. Figure S1. The upregulated Th17 cells and downregulated Tregs in peripheral blood of psoriatic patients. a The proportion of CD4+IL17 + T cells in peripheral blood of psoriatic patients and healthy controls by flow cytometry (n=10 per group, p<0.001). b The proportion of CD25+Foxp3+ T cells in peripheral blood of psoriatic patients by flow cytometry (n=10 per group, p<0.01). Data are representative of three independent experiments (mean±SEM). *P<0.05, **P<0.01, ***P<0.001. Two-tailed unpaired Student’s t-test (a, b). Th17, T helper cell 17; Treg, regulatory T cell; IL, interleukin; Foxp3, Forkhead transcription factor3. [file 12967_2023_4427_MOESM1_ESM.docx]
